# Fasting substrates predict chronic kidney disease progression in CREDENCE trial patients with type 2 diabetes

**DOI:** 10.1172/jci.insight.180637

**Published:** 2024-12-20

**Authors:** Ele Ferrannini, Simona Baldi, Maria Tiziana Scozzaro, Giulia Ferrannini, Michael K. Hansen

**Affiliations:** 1 CNR (National Research Council) Institute of Clinical Physiology, Pisa, Italy.; 2 Department of Clinical and Experimental Medicine, University of Pisa, Pisa, Italy.; 3 Department of Medicine Solna, Karolinska Institutet, Stockholm, Sweden.; 4 Internal Medicine Unit, Södertälje Hospital, Stockholm, Sweden.; 5 Janssen Research & Development, LLC, Raritan, New Jersey, USA.

**Keywords:** Cardiology, Endocrinology, Diabetes, Drug therapy, Intermediary metabolism

## Abstract

**BACKGROUND:**

Sodium-glucose cotransporter 2 inhibitors slow down progression of chronic kidney disease (CKD). We tested whether the circulating substrate mix is related to CKD progression and cardiovascular outcomes in patients with type 2 diabetes (T2D) and albuminuric CKD in the CREDENCE trial.

**METHODS:**

We measured fasting substrates in 2,543 plasma samples at baseline and 1 year after randomization to either 100 mg canagliflozin or placebo and used multivariate Cox models to explore their association with CKD progression, heart failure hospitalization/cardiovascular death (hHF/CVD), and mortality.

**RESULTS:**

Higher baseline lactate and free fatty acids (FFAs) were independently associated with a lower risk of CKD progression (HR = 0.73 [95% CI: 0.54–0.98] and HR = 0.67 [95% CI: 0.48–0.95], respectively) and hHF/CVD HR = 0.70 [95% CI: 0.50–0.99] and HR = 0.63 [95% CI: 0.42–0.94]). Canagliflozin led to a rise in plasma FFAs, glycerol, β-hydroxybutyrate, and acetoacetate. Changes in substrate between baseline and year 1 predicted an approximately 30% reduction in relative risk of both CKD progression and hHF/CVD independently of treatment. More patients who did not respond to canagliflozin treatment in terms of CKD progression belonged to the bottom lactate and FFA distribution tertiles.

**CONCLUSION:**

In T2D patients with albuminuric CKD, basic energy substrates selectively influenced major long-term endpoints; canagliflozin treatment amplified their effects by chronically raising their circulating levels.

## Abstract 2

New drug treatments for patients with heart and kidney disease and diabetes might beneficially act on the balance between basic energy substrates.

## Graphical abstract

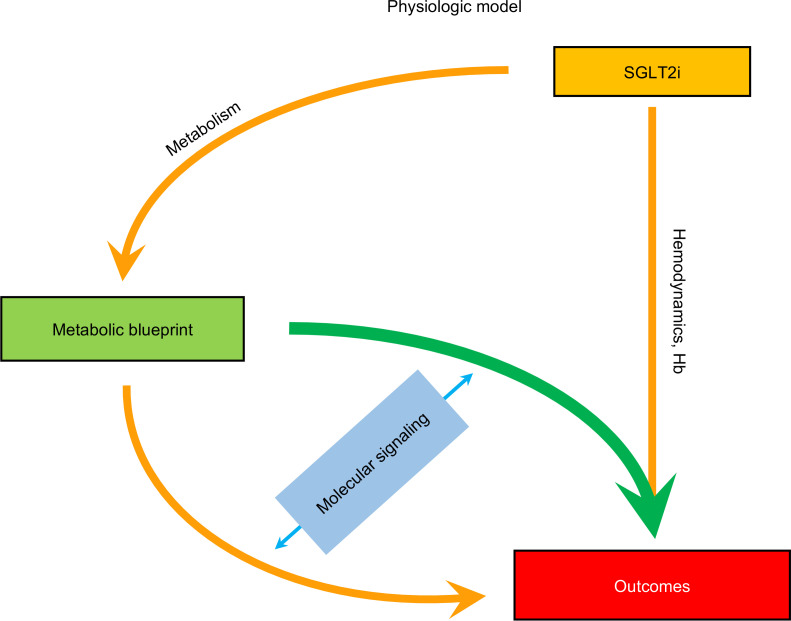

## Introduction

Sodium-glucose cotransporter 2 inhibitors (SGLT2i) in patients with type 2 diabetes (T2D) have consistently demonstrated renal and cardiovascular (CV) benefit, the latter especially for heart failure–related (HF-related) events (1–6). Since the magnitude of risk reduction cannot be fully explained by the amelioration of traditional CV risk factors, including better glycemic control, lower blood pressure, and weight loss, other mechanisms have been hypothesized (7). The shift in plasma substrate utilization from carbohydrates to lipids (i.e., free fatty acids [FFAs] and ketones) induced by SGLT2 inhibition via their glycosuric effect has been hypothesized to play a major role, as it possibly improves myocardial energy production and transduction, thereby delaying or preventing hospitalizations for HF (hHF) (8–16). A recent secondary analysis of the canagliflozin cardiovascular assessment study (CANVAS) trial, a cardiovascular outcome trial randomizing T2D patients to either canagliflozin or placebo, has indeed shown that higher fasting plasma FFAs at baseline were independently associated with 50% relative risk reduction of hHF (17). Interestingly, FFAs were essentially stable during the trial and the impact of randomized treatment on their concentrations was consistent (17).

The association between such metabolic setup and renal protection is well supported by preclinical data (18, 19). A small study in 33 patients with T2D and albuminuria showed that dapagliflozin treatment specifically increased urinary metabolites linked to improved mitochondrial function, among others β-hydroxybutyrate (β-OH) and acetoacetate (AcAc), suggesting kidney-specific ketogenesis and potential protective effects (20).

However, evidence from large clinical studies on the effect of the shift in plasma substrate utilization on renal events is lacking, as is its possible link with improved myocardial function specifically in patients with diabetic kidney disease, not the least because of the primary action of SGLT2i on the kidney.

We therefore measured major circulating substrates in samples from the canagliflozin and renal events in diabetes with established nephropathy clinical evaluation (CREDENCE) trial, which tested the effect of 100 mg canagliflozin versus placebo on renal and CV outcomes in patients with T2D and albuminuric chronic kidney disease (CKD) (6). In particular, we assessed plasma levels of glucose, lactate, FFAs, glycerol, β-OH, and AcAc to investigate their associations with outcomes (21).

## Results

Samples from 1,287 participants in the treatment arm and 1,256 in the placebo arm at baseline and at year 1 were analyzed, totaling 60% of the original cohort (Reduced CREDENCE cohort). Overall, there were 850 males and 437 females in the canagliflozin arm, and 836 males and 420 females in the placebo group (*P* = 0.783). Among the measured plasma substrates, the strongest associations were between FFAs and glycerol (*r* = 0.33 at baseline and *r* = 0.66 at year 1, *P* < 0.0001 for both), and between β-OH and AcAc (*r* = 0.44 at baseline and *r* = 0.46 at year 1, *P* < 0.0001 for both). Intraindividual correlations for each substrate over time (baseline and 1 year) were remarkably robust (with *r* values ranging from 0.40 to 0.60) regardless of treatment assignment.

### Renal endpoint versus hHF.

In the Reduced CREDENCE cohort, the effect of canagliflozin treatment on the primary composite endpoint (*n* = 330 first events) closely reproduced the result of the Full CREDENCE cohort (*n* = 585 first events) (Supplemental Figure 1; supplemental material available online with this article; https://doi.org/10.1172/jci.insight.180637DS1). In the full dataset, time to first composite renal endpoint was significantly (*P* < 0.0001) longer (707 [IQR 420] days) than time to first hHF (556 [IQR 438] days). Risk of first composite renal endpoint was significantly greater in participants who had had a first episode of hHF (*n* = 87) as compared with those who had not (*n* = 143, HR 2.92 [95% CI: 2.16–3.94], *P* < 0.0001).

### Baseline substrates versus outcomes.

**Figure 1:**
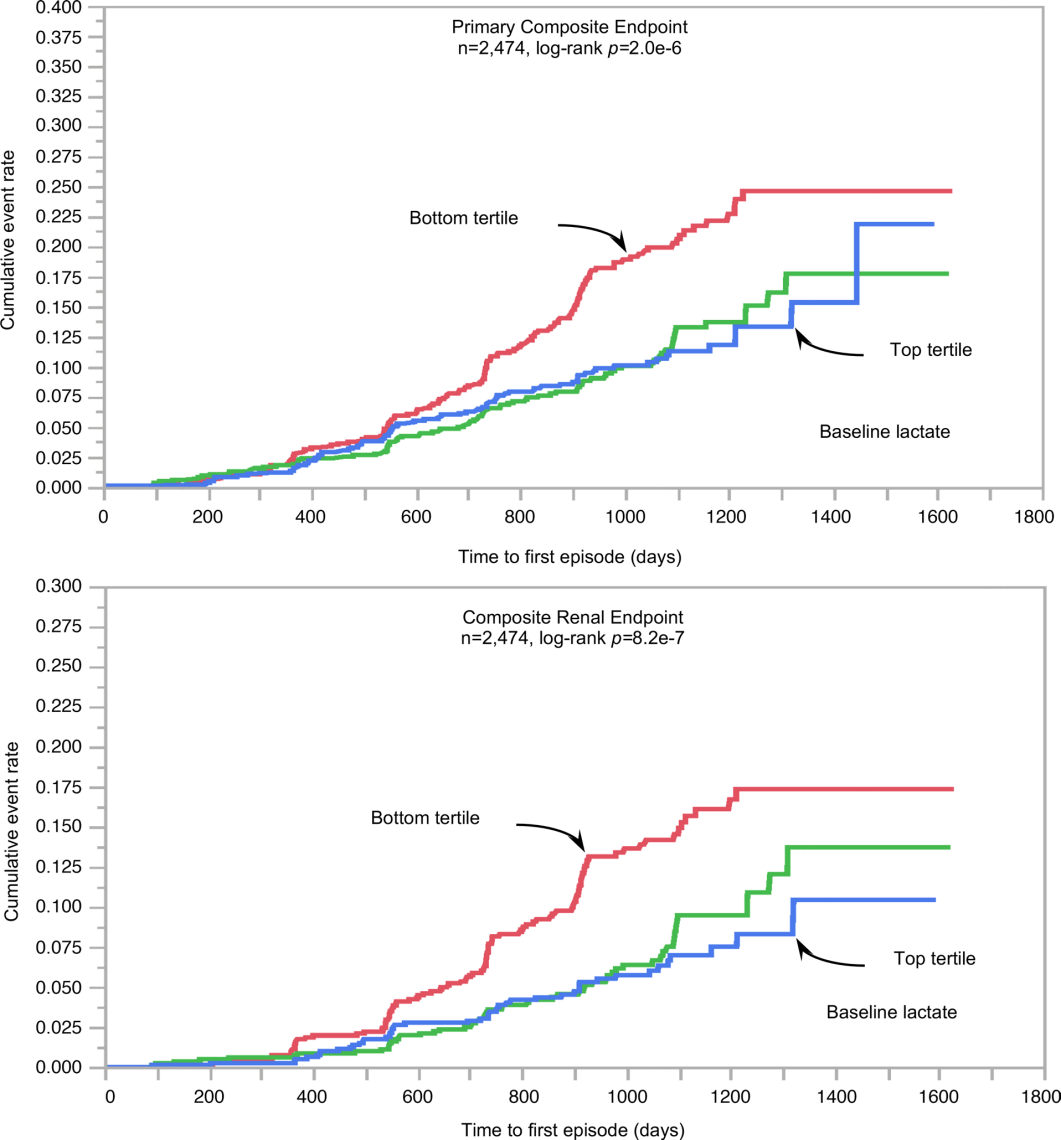
Baseline substrates versus outcomes. Kaplan-Meier plot of time to first primary composite endpoint (top) and time to composite renal endpoint (bottom) by tertile of baseline fasting plasma lactate concentrations.

In univariate Cox analysis, participants in the bottom tertile of baseline fasting plasma lactate concentrations were at significantly higher risk for both the primary composite and the renal composite endpoint (Figure 1). In multivariate Cox models using all 6 measured substrates (Model I), lactate was inversely associated with both the primary endpoint and the renal endpoint; additionally, lactate was negatively associated with all-cause death (Table 1). Notably, lactate was inversely associated with each of the individual components of the composite renal endpoint (Table 2).

**Figure 2:**
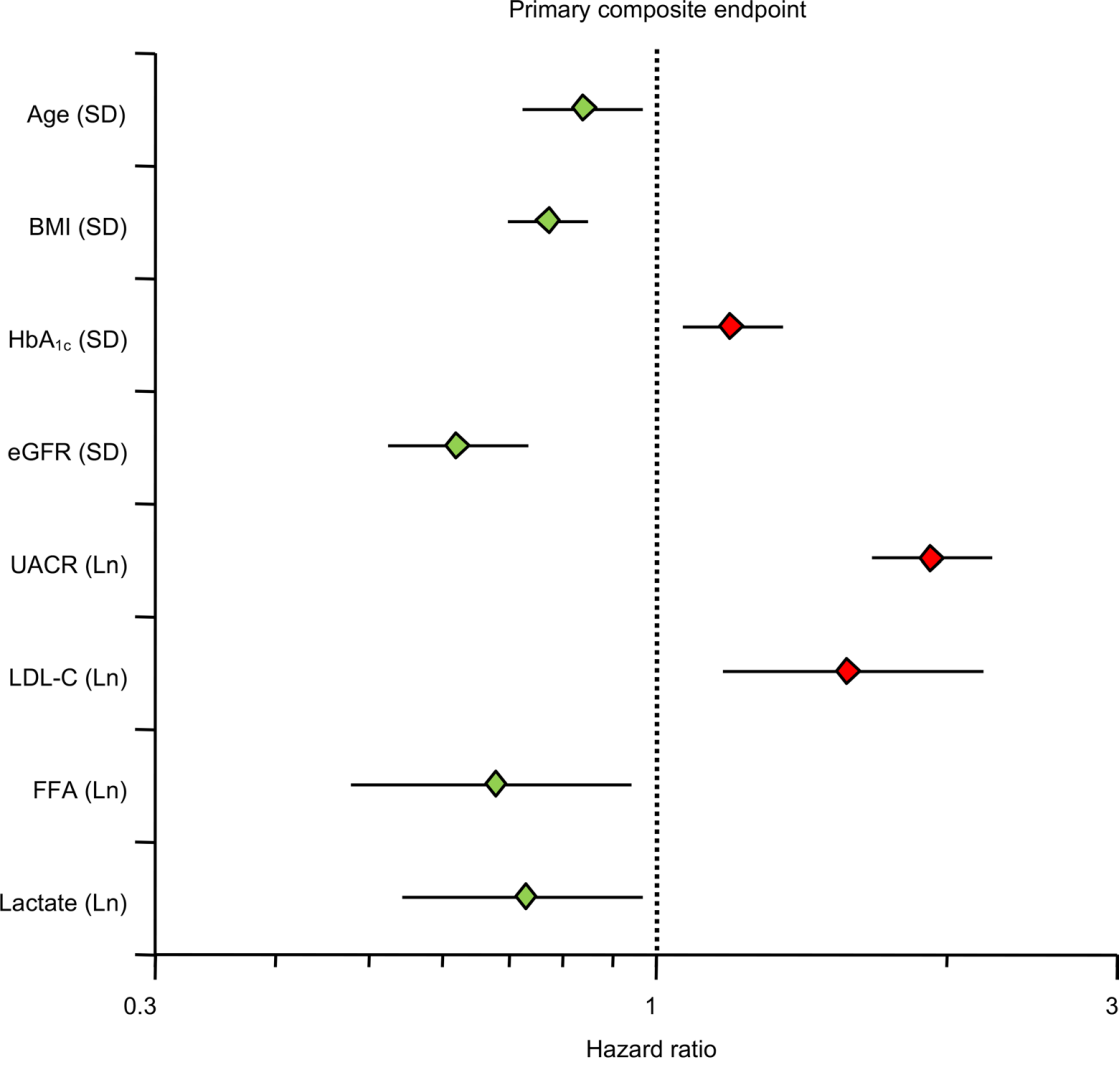
Forest plot showing the multivariate Cox proportional hazards model of the association between several covariates, including baseline fasting plasma lactate and free fatty acids, with time to first primary composite endpoint. Only covariates reaching statistical significance are plotted; additional covariates with no statistically significant differences are not plotted. SD, standard deviation; Ln, natural logarithm; BMI, body mass index; HbA_1c_, glycated hemoglobin A1c; eGFR, estimated glomerular filtration rate; UACR, urinary albumin-to-creatinine ratio; LDL-C, low-density lipoprotein cholesterol; FFA, free fatty acids.

When Model I was adjusted for the full set of covariates and potential confounders (Model II), lactate still emerged as a negative predictor of the primary composite endpoint, hHF/CVD, and all-cause death (Table 1 and Figure 2).

**Figure 3:**
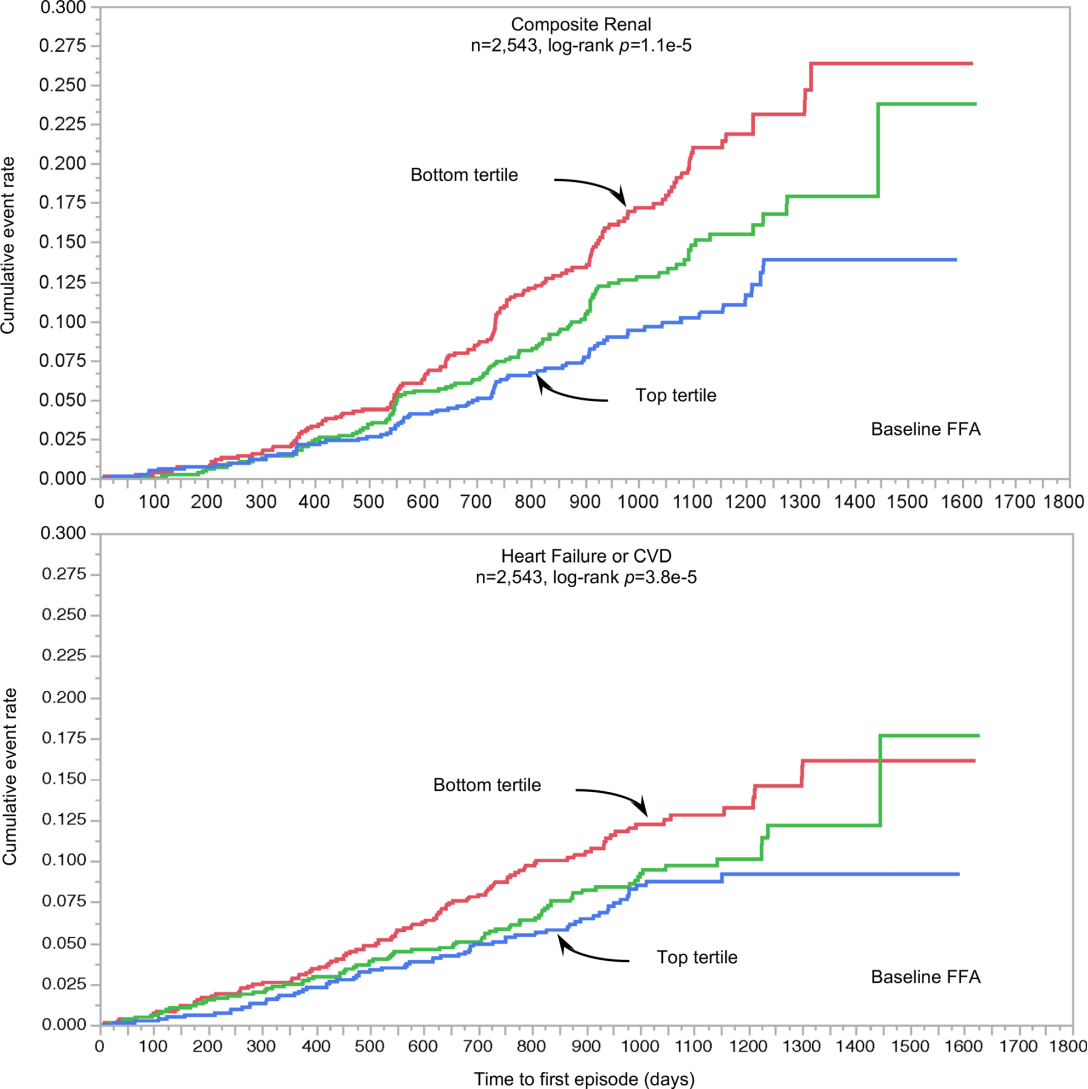
Kaplan-Meier plots of time to first composite renal endpoint and time to first hospitalized congestive heart failure by tertile of baseline fasting plasma FFA concentrations.

**Figure 4:**
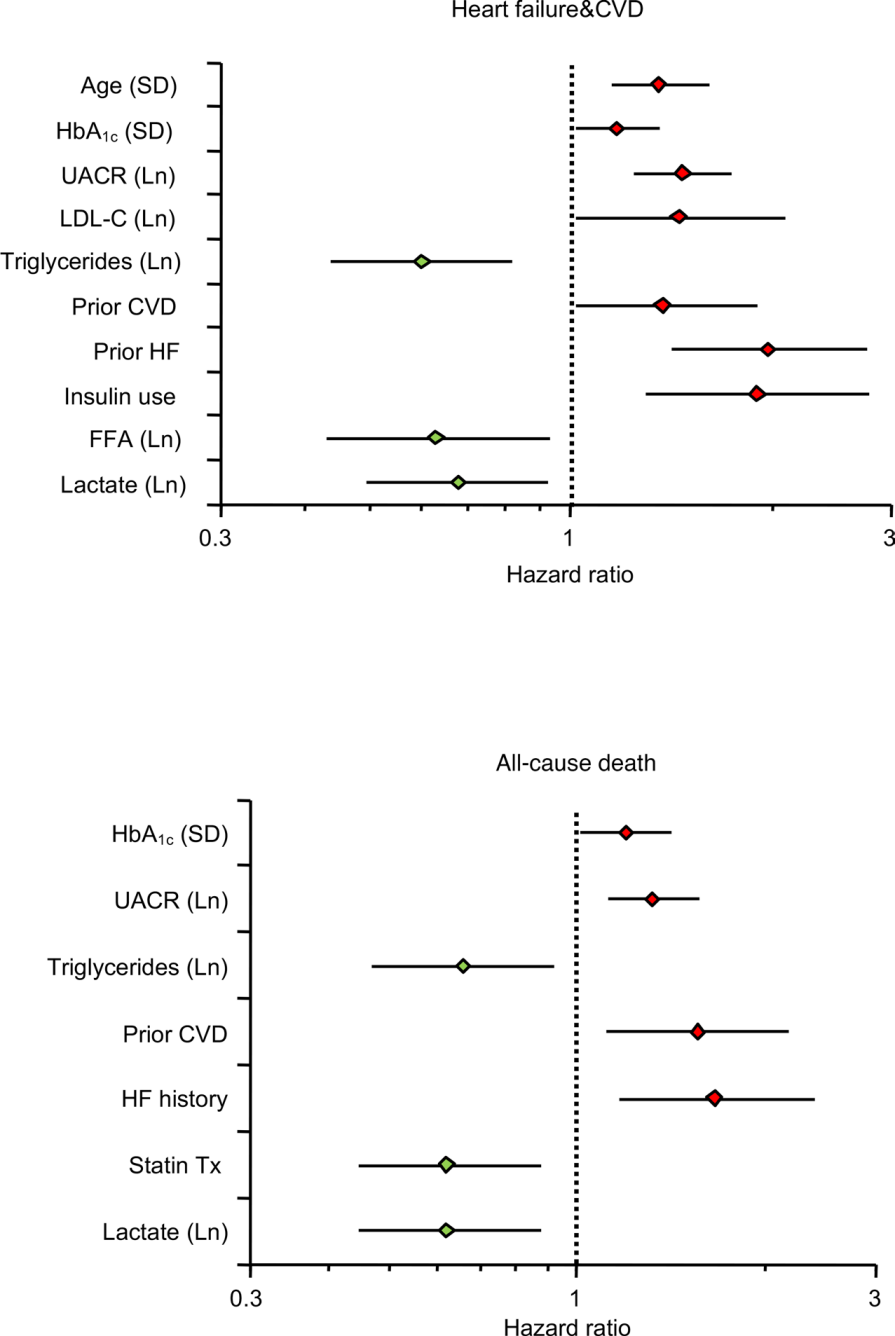
Forest plots showing the multivariate Cox proportional hazards model of the association between several covariates, including baseline fasting plasma lactate and free fatty acids, with time to first of the composite of hospitalized congestive heart failure (upper panel) or cardiovascular death and time to all-cause death (lower panel). Only covariates reaching statistical significance are plotted; additional covariates with no statistically significant differences are not plotted. SD, standard deviation; Ln, natural logarithm; HbA_1c_, glycated hemoglobin A1c; UACR, urinary albumin-to-creatinine ratio; LDL-C, low-density lipoprotein cholesterol; CVD, cardiovascular disease; HF, heart failure; FFA, free fatty acids.

In the present study cohort, patients with first on-trial hHF had a 3-fold higher history of HF (33% vs. 12%, *P* < 0.0001) and lower baseline FFA (398 [IQR 334] vs. 462 [IQR 340] μmol/L, *P* = 0.0031) than patients without this outcome. Most notably, baseline fasting plasma FFAs showed essentially the same pattern of associations as plasma lactate levels, namely, a graded increase in risk of the composite renal endpoint and hHF/CVD across decreasing concentration tertiles in univariate Cox analysis (Figure 3), and an independent negative association with the primary and renal endpoints and hHF/CVD in the fully adjusted Cox model (Table 1 and Figure 4). In the latter analysis, however, FFAs were not significant predictors of all-cause death. Along with lactate, plasma FFAs too were significant independent predictors of the components of the composite renal endpoint (Table 2).

To identify the phenotype of participants with different baseline lactate or FFA levels, we compared clinical and metabolic features across tertiles of the 2 substrates (Table 3). For the lowest versus the highest FFAs, participants were more often males, slightly younger, and with a longer duration of diabetes. The urinary albumin-to-creatinine ratio (UACR) was higher and other substrates (glucose, glycerol, β-OH, and AcAc) were lower, as was a prior history of HF. Of note, background insulin use was much higher, while metformin or sulfonylurea use was lower. For lactate, the pattern was remarkably overlapped with that for FFAs, except for a lesser use of statins and anti-thrombotics.

### Treatment.

In univariate analysis, treatment led to significant protection not only against the primary composite endpoint (Supplemental Figure 1), but also against the composite renal endpoint (HR 0.68 [95% CI: 0.51–0.89]) and its components (HR 0.69 [95% CI: 0.50–0.95]) for CV and renal death, HR 0.59 [95% CI: 0.43–0.80] for serum creatinine doubling, HR 0.69 [95% CI: 0.50–0.95] for end-stage kidney disease, and HR 0.56 [95% CI: 0.38–0.83] for end-stage kidney disease with an estimated glomerular filtration rate (eGFR) below 15.

As compared with placebo, canagliflozin treatment led to a decrease in plasma glucose, and a rise in the plasma concentrations of FFA, glycerol, β-OH, and AcAc (Table 4). Using year 1 substrate concentrations, multivariate Cox models (Model I) indicated that higher levels of FFA and lactate were independently associated with significantly reduced risk of both the primary and the renal endpoint (Supplemental Table 1). With full adjustment for all covariates (Model II), lactate — but not FFA — was still an inverse predictor of both the primary and the renal endpoint.

In the 96 patients experiencing a first nonfatal myocardial infarction, neither FFA nor lactate (baseline or year 1 samples) was a significant predictor, nor did canagliflozin treatment protect against this outcome.

To characterize the specific impact of canagliflozin treatment on outcomes, we calculated the changes in substrate levels between baseline and year 1, and related the top quartile of such changes to main endpoints in Cox models adjusting for baseline substrate values and treatment itself. The results (Table 5) show that each substrate, except glycerol, predicted an approximately 30% reduction in relative risk of both the primary composite endpoint and hHF/CVD independently of treatment.

### Non-responder analysis.

**Figure 5:**
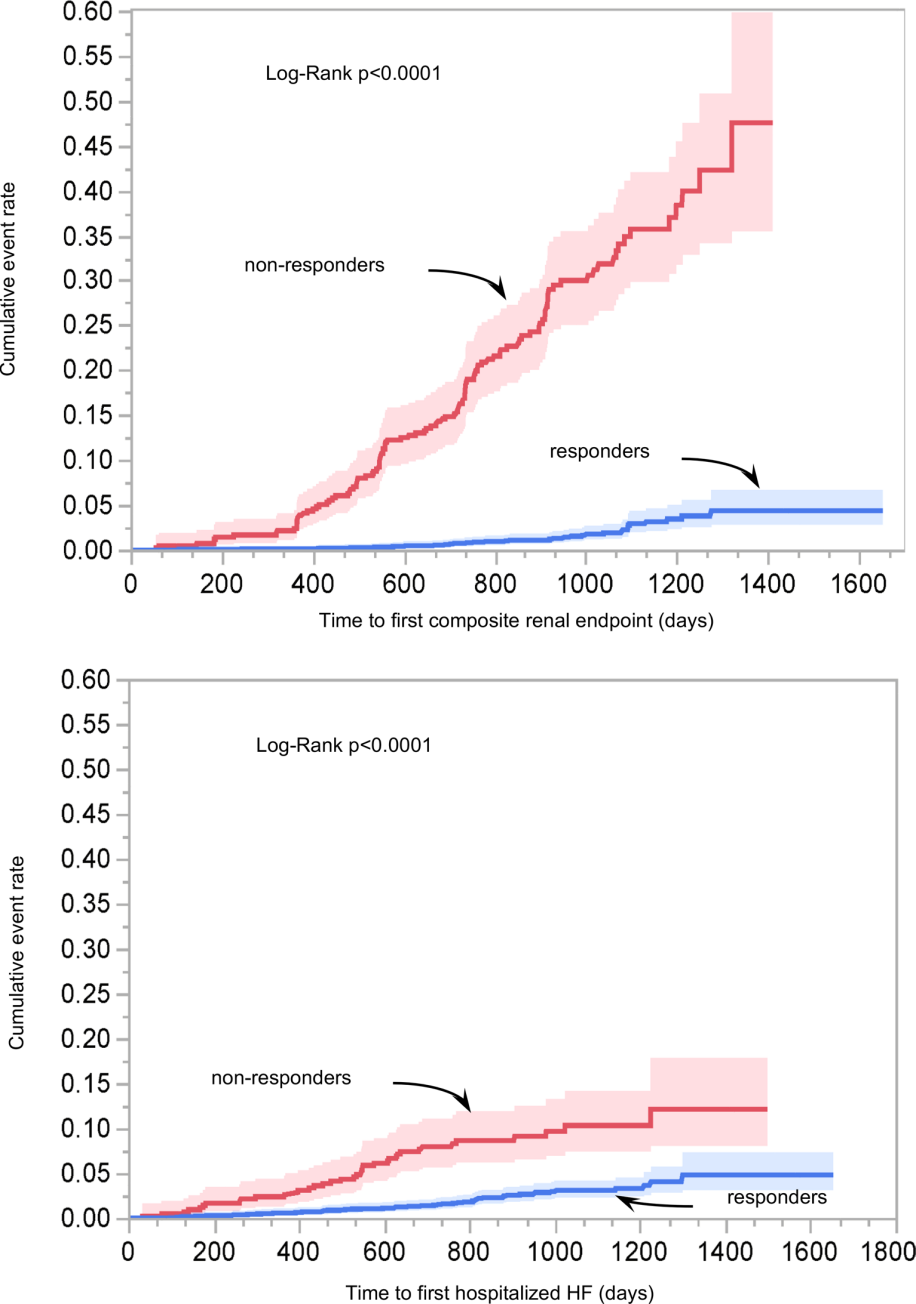
Kaplan-Meier plot of time to first composite renal endpoint and time to first hospitalized congestive heart failure by treatment responder status in the treatment arm (see text for definition of responder).

Canagliflozin significantly reduced eGFR slope (–2.0 [IQR –4.7] vs. –3.5 [IQR –6.2] mL/min/1.73 m^2^/year of placebo, *P* < 0.0001). By defining non-responders as those participants in the drug treatment arm falling in the top quartile of the slope distribution (i.e., an eGFR decline greater than –4.77 mL/min/1.73 m^2^/year), non-responders had a much higher risk of composite renal, but also a higher risk of hHF (Figure 5). Including both non-responder status and drug treatment in a multi-adjusted Cox model for the composite renal as well as the full list of risk factors, the multi-adjusted HR was 9.79 [95% CI: 7.40–12.95] for the former and 0.94 [95% CI: 0.76–1.16] for the latter. For the hHF endpoint, non-responder status had an HR of 2.52 [95% CI: 1.89–3.35], while drug treatment had an HR of 0.68 [95% CI: 0.52–0.90]; in neither model was there a significant interaction between responder status and drug treatment. There were 29% non-responders in the bottom baseline lactate third versus 23% in the top third (*P* = 0.0110); likewise, there were 30% non-responders in the bottom baseline FFA third versus 21% in the top third (*P* = 0.0004).

## Discussion

Our principal finding is that, in high-risk patients with T2D and albuminuric CKD, fasting plasma lactate concentrations measured before randomization were consistent, strong predictors of renal outcomes (primary composite, renal composite, and its components), hHF/CVD and all-cause mortality, independently of other circulating substrates, anthropometrics, and conventional risk factors (Figure 2). Lower lactates were associated with a 30%–40% risk increase for the renal endpoint, emerging roughly 18 months after randomization (Figure 1) regardless of treatment assignment. Participants in the lowest tertile of lactate distribution also had lower levels of all the other measured circulating substrates, with a longer duration of diabetes, but better glycemic control, lower eGFR, and higher AUCR (Table 2). Incidentally, this group with more advanced CKD were receiving less statin and anti-thrombotic therapy despite their high CV risk, and more insulin than metformin for their hyperglycemia.

Importantly, baseline fasting FFAs also predicted renal outcomes in a similar fashion as lactate in addition to predicting hHF/CVD in fully adjusted models (Table 1). The hHF/CVD result confirms the findings previously reported in the CANVAS population (17). The extended phenotype of participants in tertiles of baseline FFAs essentially overlays that of lactate except for the use of CV drugs (Table 2). The pattern of significant risk predictors extracted from the multivariate analysis highlights the protective effect of higher levels of both substrates against the primary composite endpoint in the face of powerful negative factors, especially albuminuria (Figure 2).

Another salient result of the present study is the effect of canagliflozin treatment. The lipolysis couple, i.e., FFAs and glycerol, and the ketone couple, i.e., β-OH and AcAc, both rose in response to SGLT2 inhibition, as expected and previously shown also in CANVAS (17). Treatment-induced changes in circulating substrates measured at year 1 can be confidently referred to as a very early time after randomization because similar changes have been previously shown to occur already following the first drug dose and to stabilize thereafter (8, 9). Furthermore, large changes in either plasma lactate or FFAs between baseline and year 1 were significantly associated with an approximately 30% reduction in relative risk of both the primary composite endpoint and hHF/CVD independently of treatment.

**Figure 6:**
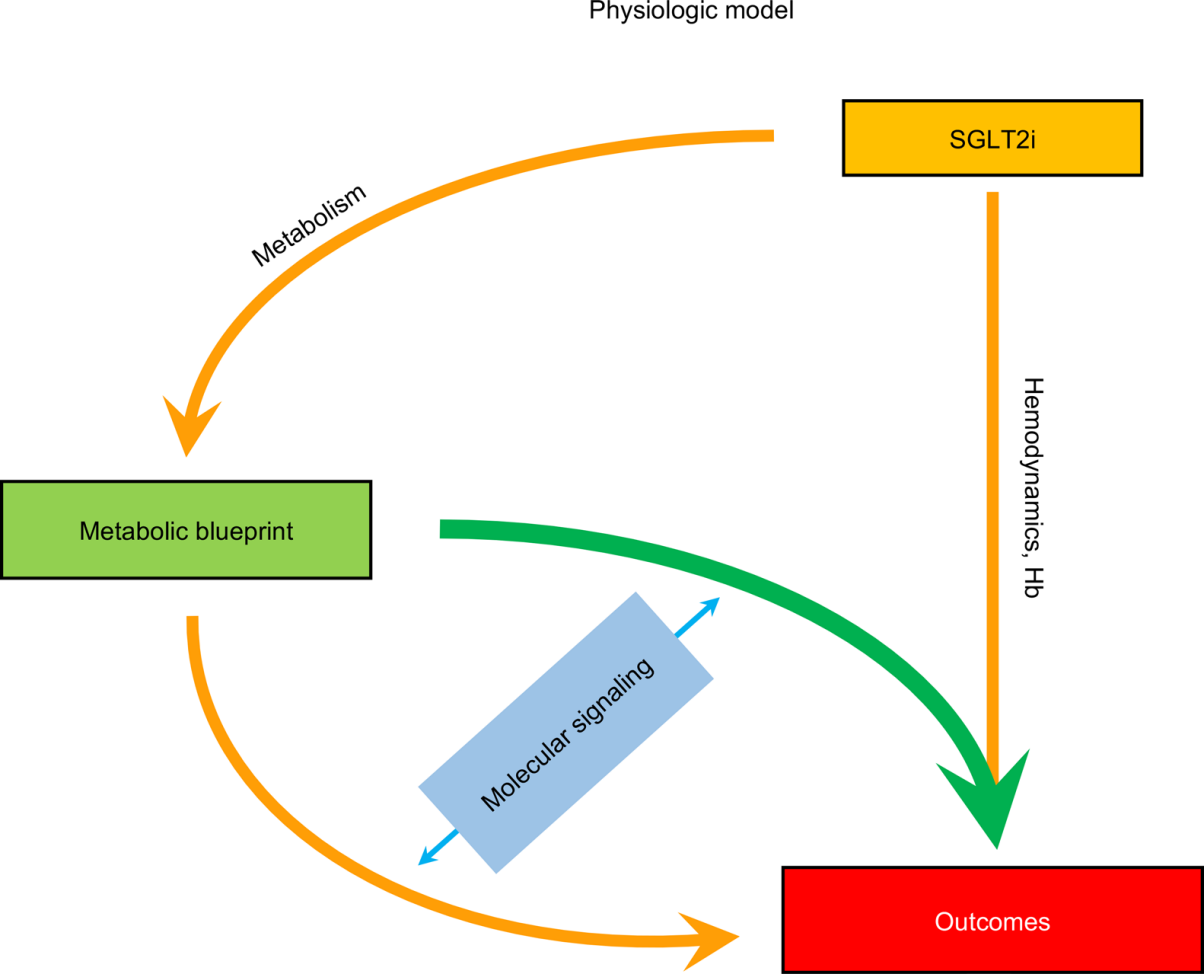
Organization of current results into a minimal physiologic model. A constitutive metabolic blueprint influences long-term cardio-renal outcomes; the metabolic changes that follow SGLT2 inhibition essentially reinforce the constitutive pattern, while hemodynamic and oxygen-delivery effects exert their own influence on outcomes. Subserving molecular pathways (enzyme activities, signaling, redox shifts, etc.) are still under study.

A coherent physiologic interpretation of these findings is that, via daily recurring urine glucose loss, SGLT2 blockade causes a shift from carbohydrate to fat utilization, which at least in some individuals reinforces an extant, constitutive metabolic blueprint (Figure 6) (22). The increased supply of energy-rich substrates arguably translates into protection of high-energy output organs — such as the heart and the kidney — on the way to failure (10, 11). This protection is extensively reflected by the beneficial class effects of SGLT2i on the heart and the kidney which, after the landmark cardiovascular outcome trials in patients with T2D, are now established even in patients with HF and without T2D (23, 24) and in patients with CKD regardless of the presence of T2D (25, 26). In the present study, patients with first on-trial hHF had lower baseline FFAs than patients without this outcome. As the heart runs predominantly on FFAs, higher baseline FFAs were in some degree protective against HF/CVD, and canagliflozin treatment was replaced by year 1 FFA levels in statistical models of prediction. SGLT2-induced changes in hemodynamics (e.g., higher hematocrit, lower blood pressure, decreased interstitial fluid accumulation and oxygen delivery, e.g., higher blood hemoglobin) plausibly concur with the metabolic shifts to influence cardio-renal outcomes (Figure 6) (27, 28).

Subsequently, multiple SGLT2i trials converged in showing substantial protection against progression of renal impairment (which was the primary endpoint in CREDENCE) (6). The kidney consumes energy at a resting rate (~50 cal/min/100 g) that is 5 times that of the heart. Proximal tubules are rich in mitochondria and gluconeogenic enzyme activities and use mostly FFAs for energy production, while the distal tubules and collecting ducts have a high glycolytic capacity (29). In the present study, patients reaching a first on-trial composite renal endpoint had lower baseline eGFR (46 ± 16 vs. 58 ± 18 mL/min/1.73 m^2^, *P* < 0.0001), higher baseline UACR (2442 [IQR 2252] vs. 838 [IQR 1136] mg/g, *P* < 0.0001), lower baseline lactate (1.68 [IQR 0.98] vs. 2.00 [IQR 1.10] mmol/L, *P* < 0.0001) and FFAs (372 [IQR 320] vs. 475 [IQR 340] μmol/L, *P* < 0.0001) than patients without this outcome. Thus, both baseline lactate and FFAs and their changes following SGLT2i treatment turn out to relate to the progression of renal damage. How lactate may influence renal function in addition to energy provision is presently uncertain. Because the kidney can both make and utilize lactate, lower circulating concentrations may reflect decreased release from a dysfunctional kidney or reduced supply to the kidney, either way a signature of net shortfall of this substrate for utilization (30). There is also the alternate hypothesis that increased lactate production under diabetic conditions is a marker of and contributor to mitochondrial dysfunction, becoming a feed-forward component to diabetic kidney disease pathogenesis (31, 32).

In summary, the present analysis demonstrates that in patients with CKD and high CV risk there is a constitutive circulating metabolite blueprint whereby basic energy substrates selectively influence major organ endpoints, such as renal function and cardiac contractility, but not ischemic heart disease, with remarkable consistency — i.e., independence from traditional risk factors — and power — i.e., strong reductions in relative risk. SGLT2 inhibition amplifies the effect of FFAs by chronically raising their circulating levels.

The present study has obvious limitations in the design, in that it is a secondary analysis of the CREDENCE trial, not including the whole cohort, and results might not be generalizable to patients without CKD and/or T2D. There are also limitations to the interpretation of single fasting plasma samples: (a) circulating substrate levels reflect handling in multiple organs, (b) concentrations and not fluxes were measured, and (c) amino acids, which are poor oxidative substrates for the heart but key agonists in multiple renal functions (30, 33), were not measured. Importantly, the potential mechanistic connections between plasma levels and outcomes we outline do not in any way conflict with the operation of different mechanisms (e.g., hemodynamic) (34) of cardio-renal protection by SGLT2i. In fact, the pattern of substrate associations we describe is the result of stringent filtering of signals by extensive statistical adjustment and no adjustment was made for multiple comparisons, nor can we rule out the possibility of residual confounding. On the other hand, weaker, but nonetheless, important signals may be lost in the process.

Coherent integration of circulating and excretion signatures into a general metabolic portrait incorporating SGLT2i pharmacodynamics is a daunting task for future research, toward which our analyses, current and previous (17, 28), offer solid insight.

## Methods

### Sex as a biological variable.

Both males and females fulfilling the inclusion criteria were enrolled in the CREDENCE trial.

The CREDENCE trial enrolled 4,401 patients with T2D, CKD (eGFR ≥ 30 to < 90 mL/min/1.73 m^2^) and albuminuria (UACR > 300 to ≤ 5,000 mg/g) who were randomized (1:1) to 100 mg canagliflozin or placebo (Full CREDENCE cohort) (6). The primary composite outcome was end-stage kidney disease (dialysis for at least 30 days, kidney transplantation, or an eGFR of <15 mL/min/1.73 m^2^ sustained for at least 30 days, doubling of serum creatinine level from baseline sustained for at least 30 days, or death from kidney or CV disease). Secondary outcomes included (a) a composite of end-stage kidney disease, doubling of serum creatinine level, and death because of kidney disease; (b) a composite of CV death (CVD) or hHF; (c) hHF alone; and (d) death from any cause.

Details of the trial design and oversight, participants, inclusion and exclusion criteria, randomization, treatment, follow-up, and outcomes have been published (4) (ClinicalTrials.gov NCT02065791). CREDENCE was stopped early after a planned interim analysis, with a final median follow-up of 2.6 years.

Plasma samples were obtained from participants who consented to have blood samples taken for exploratory biomarker research wherever local regulations permitted. All the samples were analyzed and no retrospective sample selection was done. Plasma aliquots were stored at –80°C and, in October 2021, were transferred to the Metabolism Unit of the Department of Clinical and Experimental Medicine at University of Pisa, Italy for analysis. Paired samples from the baseline and year 1 collection were assayed in the same run; lab operators were blinded to the patients’ data.

### Analytical methods.

Plasma glucose, lactate, FFAs, glycerol, β-OH, and AcAc were measured by in-house automated spectrophotometric enzymatic methods on a Beckman UniCel DXC600 Synchron Analyzer (5, 6). Within-assay and between-assay coefficients of variation ranged between <1% and <7% for all.

### Statistics.

Data are presented as mean ± SD or median (IQR) for continuous variables showing a non-normal distribution (by Shapiro-Wilk test). Baseline characteristics across tertiles of lactate and FFAs are presented as mean ± SD or median (IQR). Group comparisons were analyzed by χ^2^ statistics (for nominal variables), Student’s *t* test, or Wilcoxon’s test for normally and non-normally distributed variables, respectively. Paired data (baseline and year 1) by treatment (canagliflozin or placebo) were analyzed by repeated-measures ANOVA. Univariate correlations were tested by Spearman’s *r*. Cumulative event rates for the time-to-first event were computed by the Kaplan-Meier estimator and compared by the log-rank test. Cox proportional hazards models were used to test the association of predictors with endpoints in a stepwise fashion. First, all 6 measured substrates (glucose, lactate, FFAs, glycerol, β-OH, and AcAc) were used in multivariate Cox models (Model I); next, Model I Cox models were further adjusted for sex, age, body mass index (BMI), smoking, eGFR, HbA_1c_, systolic blood pressure, prior CV disease, HF at baseline, UACR, high-density lipoprotein cholesterol (HDL), low-density lipoprotein cholesterol (LDL), triglycerides, and use of statins, anti-thrombotics, loop and/or non-loop diuretics, beta blockers, metformin, sulphonylureas, insulin, and glucagon-like peptide-1 receptor agonists (GLP-1Ras) (Model II); continuous variables with non-normal distribution (substrates, UACR, lipids) were transformed to their natural logarithms. For year 1 data, year 1 plasma substrate concentrations were used, and in the Model II Cox model treatment was added as a further covariate. Results are expressed as HR and 95% CI. Individual eGFR slopes were calculated by simple regression of values recorded between baseline and 156 weeks, with the first postrandomization time at 13 weeks; participants falling in the top quartile of the slope distribution in the drug treatment arm were categorized as non-responders. Cumulative event rates for the time to primary outcome and to first hHF in responders versus non-responders were computed by the Kaplan-Meier estimator and compared by the log-rank test. A multivariable Cox regression model, including both responder status and drug treatment and their interaction in addition to all other covariates, was run. All analyses were carried out using JMP 16.2.0 (https://www.jmp.com/en_us/home.html).

### Study approval.

CREDENCE was approved by the ethics committees at each site (ClinicalTrials.gov NCT02065791). All participants provided written informed consent.

### Data availability.

The data sharing policy of Janssen Pharmaceutical Companies of Johnson & Johnson is available at https://www.janssen.com/clinical-trials/transparency As noted on this site, requests for access to the study data can be submitted through Yale Open Data Access (YODA) Project site at http://yoda.yale.edu No applicable resources were generated or analyzed during the current study. Raw data are available in the Supporting Data Values file.

## Author contributions

EF conceptualized and designed this study, acquired and analyzed data, and wrote and revised the manuscript. SB and MTS acquired and analyzed data and revised the manuscript. GF interpreted data and wrote and revised the manuscript. MKH was the main investigator of the CREDENCE trial, responsible for data acquisition, analysis, and manuscript revision for this work.

## Supplementary Material

Supplemental data

ICMJE disclosure forms

Supporting data values

## Figures and Tables

**Table 5:**
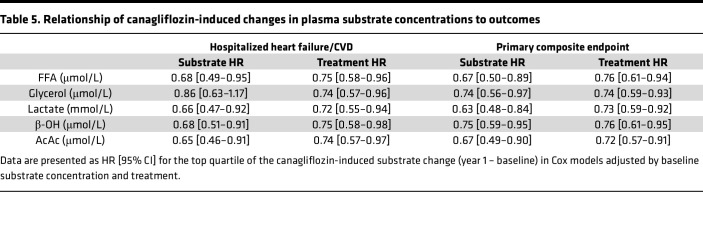
Relationship of canagliflozin-induced changes in plasma substrate concentrations to outcomes

**Table 1:**
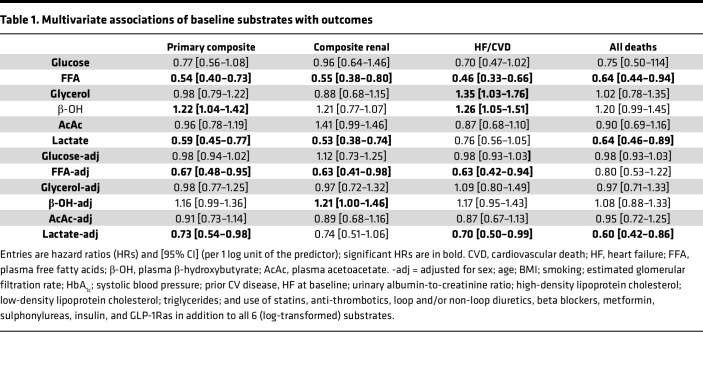
Multivariate associations of baseline substrates with outcomes

**Table 2:**
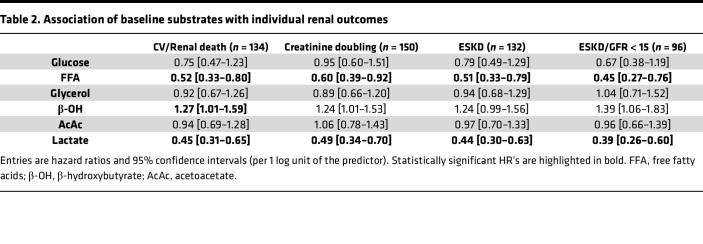
Association of baseline substrates with individual renal outcomes

**Table 3:**
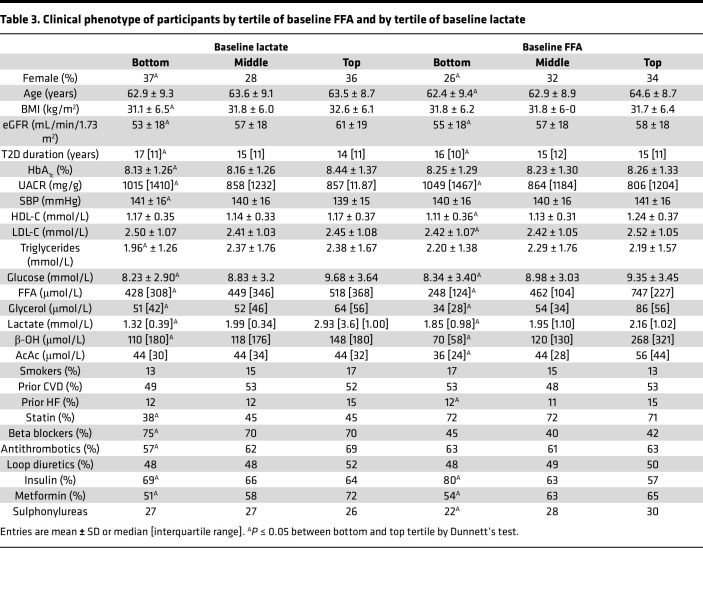
Clinical phenotype of participants by tertile of baseline FFA and by tertile of baseline lactate

**Table 4:**
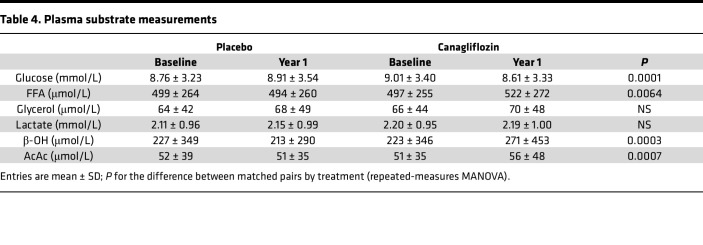
Plasma substrate measurements

## Version 1 12/20/2024

Electronic publication
