# Supplementary material for: Fasting substrates predict chronic kidney disease progression in CREDENCE trial patients with type 2 diabetes
Source: JCI Insight. 2024 Dec 20;9(24):e180637. doi: 10.1172/jci.insight.180637 (PMC11665565; doi:10.1172/jci.insight.180637)

Supplemental material

Supplemental Table 1. Multivariate association of Year 1 Substrates with Outcomes. §

	Primary Comp.	Comp. Renal	HF/CV death	All deaths
**Glucose**	0.77 [0.53–1.12]	**0.62 [0.61–0.97]**	1.12 [0.72–1.76]	1.27 [0.76–2.13]
**FFA**	**0.62 [0.44–0.89]**	0.71 [0.47–1.08]	**0.43 [0.29–0.66]**	**0.53 [0.33–0.87]**
**Glycerol**	0.95 [0.74–1.21]	0.93 [0.70–1.23]	1.27 [0.94–1.70]	0.99 [0.71–1.38]
**β-OH**	**1.22 [1.02–1.46]**	1.13 [0.92–1.39]	**1.37 [1.10–1.71]**	**1.38 [1.07–1.78]**
**AcAc**	**0.72 [0.58–0.88]**	**0.72 [0.56–0.92]**	**0.74 [0.58–0.95]**	0.80 [0.59–1.07]
**Lactate**	**0.40 [0.30–0.55]**	**0.35 [0.24–0.50]**	0.71 [0.49–1.04]	**0.60 [0.39–0.93]**
**Treatment**	**0.58 [0.44–0.77]**	**0.45 [0.32–0.62]**	082 [0.59–1.13]	1.04 [0.71–1.52]
**Glucose-adj.**	**0.70 [0.49–1.00]**	**0.60 [0.40–0.91]**	0.97 [0.62–1.52]	1.16 [0.68–1.99]
**FFA-adj.**	0.78 [0.54–1.14]	0.91 [0.58–1.41	**0.58 [0.37–0.92]**	0.75 [0.44–1.28]
**Glycerol-adj.**	0.87 [0.65–1.17]	0.82 [0.58–1.15]	1.00 [0.70–1.43]	0.86 [0.58–1.29]
**β-OH-adj.**	**1.23 [1.04–1.46]**	**1.22 [1.00–1.48]**	**1.28 [1.02–1.62]**	1.22 [0.95–1.58]
**AcAc-adj.**	**0.75 [0.61–0.92]**	**0.74 [0.58–0.94]**	**0.75 [0.58–0.98]**	0.86 [0.63–1.18]
**Lactate-adj.**	**0.45 [0.32–0.64]**	**0.39 [0.26–0.59]**	**0.66 [0.44–0.99]**	**0.60 [0.38–0.97]**

§ Entries are hazard ratios (HR) and [95% confidence intervals] (per 1 log unit of the predictor); significant HRs are in bold. CV, cardiovascular; HF, heart failure; FFA, plasma free fatty acids; β-OH, plasma β-hydroxybutyrate; AcAc, plasma acetoacetate.

-adj = adjusted for sex; age; body mass index; smoking; estimated glomerular filtration rate; HbA_{1c}; systolic blood pressure; prior CV disease, HF at baseline; urinary albumin-to-creatinine ratio; high-density lipoprotein cholesterol; low-density lipoprotein cholesterol; triglycerides; and use of statins, anti-thrombotics, loop and/or non-loop diuretics, ß-blockers, metformin, sulphonylureas, insulin, and GLP-1 Ra's in addition to all 6 (log-transformed) substrates.

Supplemental Figure 1

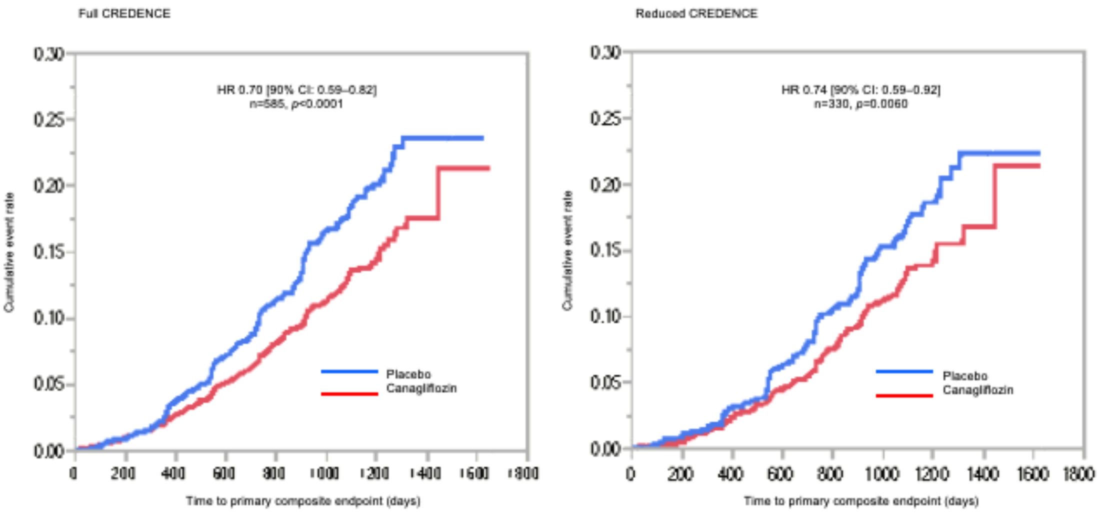

Supplement: Supplemental data [file jciinsight-9-180637-s058.pdf]
